# *Antrodia cinnamomea* induces anti-tumor activity by inhibiting the STAT3 signaling pathway in lung cancer cells

**DOI:** 10.1038/s41598-019-41653-9

**Published:** 2019-03-26

**Authors:** Tsung-Teng Huang, Ying-Wei Lan, Chuan-Mu Chen, Yun-Fei Ko, David M. Ojcius, Jan Martel, John D. Young, Kowit-Yu Chong

**Affiliations:** 1 Department of Medical Biotechnology and Laboratory Sciences, College of Medicine, Chang Gung University, Taoyuan, 33302 Taiwan; 2 Center for Molecular and Clinical Immunology, College of Medicine, Chang Gung University, Taoyuan, 33302 Taiwan; 3 Chang Gung Immunology Consortium, Chang Gung Memorial Hospital, Linkou, Taoyuan 33305 Taiwan; 4 Graduate Institute of Biomedical Sciences, Division of Biotechnology, College of Medicine, Chang Gung University, Taoyuan, 33302 Taiwan; 5 Department of Life Sciences, and Ph.D. Program in Translational Medicine, National Chung Hsing University, Taichung, 402 Taiwan; 6 The iEGG and Animal Biotechnology Center, National Chung Hsing University, Taichung, 402 Taiwan; 7 Chang Gung Biotechnology Corporation, Taipei, 10508 Taiwan; 8 Biochemical Engineering Research Center, Ming Chi University of Technology, New Taipei City, 24301 Taiwan; 9 Department of Biomedical Sciences, University of the Pacific, Arthur Dugoni School of Dentistry, San Francisco, CA 94103 USA; 10 Laboratory of Cellular Physiology and Immunology, Rockefeller University, New York, NY 10021 USA; 11 Department of Family Medicine, Chang Gung Memorial Hospital, Linkou, Taoyuan, 33305 Taiwan; 12 Centre for Stem Cell Research, Faculty of Medicine and Health Sciences, Universiti Tunku Abdul Rahman, Kajang, 43000 Selangor Malaysia

## Abstract

We examined the effects of an *Antrodia cinnamomea* ethanol extract (ACEE) on lung cancer cells *in vitro* and tumor growth *in vivo*. ACEE produced dose-dependent cytotoxic effects and induced apoptosis in Lewis lung carcinoma (LLC) cells. ACEE treatment increased expression of p53 and Bax, as well as cleavage of caspase-3 and PARP, while reducing expression of survivin and Bcl-2. ACEE also reduced the levels of JAK2 and phosphorylated STAT3 in LLC cells. In a murine allograft tumor model, oral administration of ACEE significantly inhibited LLC tumor growth and metastasis without affecting serum biological parameters or body weight. ACEE increased cleavage of caspase-3 in murine tumors, while decreasing STAT3 phosphorylation. In addition, ACEE reduced the growth of human tumor xenografts in nude mice. Our findings therefore indicate that ACEE inhibits lung tumor growth and metastasis by inducing apoptosis and by inhibiting the STAT3 signaling pathway in cancer cells.

## Introduction

Lung cancer is one of the most prevalent cancers and the leading cause of cancer-related mortality worldwide^1^. Despite recent advances made in the diagnosis and treatment of lung cancer over the past two decades, prognosis remains poor and chemotherapy provides only minimal survival gain^2^. Current chemotherapeutic drugs used to treat lung cancer produce serious side effects and may lose their efficacy due the development of drug resistance. There is therefore an urgent need for safe, effective and affordable new therapeutics to treat this disease.

Signaling transducer and activator of transcription (STAT) proteins are a family of transcription factors that include STAT1 to STAT6^3^. STAT3 is constitutively activated in a variety of cancers, including lung tumors^4,5^. STAT3 is activated via tyrosine phosphorylation, which is mediated by growth factor receptor tyrosine kinases such as epidermal growth factor receptor (EGFR), vascular endothelial growth factor receptor (VEGFR), Janus kinases (JAKs), and Src family kinases^3^. Previous studies have shown that STAT3 plays a vital role in preventing cell apoptosis and stimulating cell proliferation during tumor development^3,6^. Inhibition of STAT3 activation may thus represent an effective approach to treat lung cancer.

*Antrodia cinnamomea* (also called *Antrodia camphorata*) is a traditional medicinal mushroom from Taiwan that has been used for the treatment of diarrhea, abdominal pain, hypertension, itchy skin and cancer^7^. Studies have shown that *A. cinnamomea* possesses an extensive range of pharmacological activities, including anti-inflammatory activities^8^, hepatoprotective properties^9^, immunomodulation^10^ and antioxidant activities^11^. *A. cinnamomea* also displays anti-cancer activities against hepatocellular cancer, prostate cancer, bladder cancer and breast cancer^12–15^. A previous study showed that an ethanol extract of the mycelium induces apoptosis of A549 lung cancer cells by down-regulating expression of galectin-1, RhoGDI-α, calpain-1 small subunit and eIF-5A^16^. An ethanol extract of *A. cinnamomea* fruiting bodies has also been shown to inhibit migration of highly metastatic CL1-5 lung cancer cells by reducing expression of matrix metalloproteinase-2/9 via the mitogen-activated protein kinase (MAPK) and phosphatidylinositiol-3-kinase/Akt signaling pathways^17^. However, the molecular mechanism of the anti-cancer activity of *A. cinnamomea* in lung cancer cells has not been studied in detail. In the present study, we examined the effects and mechanism of action of an *Antrodia cinnamomea* ethanol extract (ACEE) on lung cancer cells *in vitro* and *in vivo*.

## Results

### ACEE induces apoptosis in lung cancer cells

**Figure 1:**
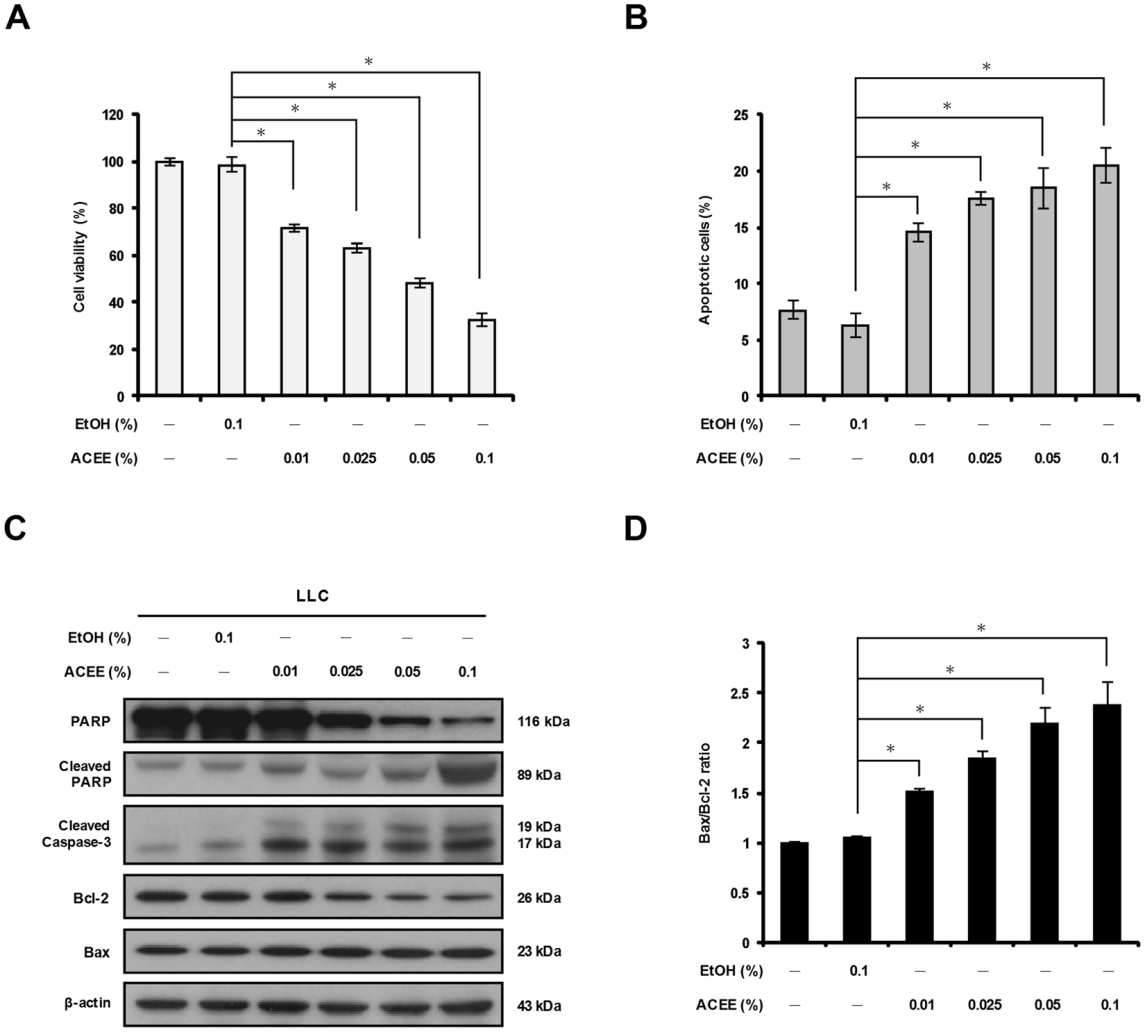
ACEE decreases viability of lung cancer cells by inducing apoptosis. (**A**) LLC cells were treated with ACEE (0.01–0.1%) for 24 h, and cell viability was monitored using the MTT assay. (**B**) ACEE induces apoptosis in LLC cells. Cells were treated with ACEE for 24 h and stained with annexin V/propidium iodide (PI) prior to flow cytometry analysis. Apoptotic cells were defined as annexin V+/PI− plus annexin V+/PI+ cells. (**C**) Western blotting of PARP, cleaved PARP, cleaved caspase-3, Bcl-2 and Bax in ACEE-treated LLC cells. β-actin was used as an internal control. (**D**) Relative Bax/Bcl-2 ratio of protein levels in ACEE-treated LLC cells was determined by densitometry. Data are presented as means ± SEM of three experiments preformed in duplicate. *P < 0.05 versus control ethanol-treated cells.

We examined whether ACEE produces cytotoxic effects on Lewis lung carcinoma (LLC) and CL1-5 lung cancer cells using the MTT cell viability assay. ACEE treatment for 24 h reduced LLC cell viability in a dose-dependent manner compared to ethanol (EtOH) used as a control (Fig. 1A, ACEE vs. EtOH). In CL1-5 cells, ACEE treatment reduced viability at 0.05 and 0.1% but produced no effect at lower concentrations (Supplementary Fig. S1B). ACEE did not reduce the viability of human MRC-5 fetal lung fibroblasts used as a control for non-cancerous cells (Supplementary Fig. S1A). ACEE treatment significantly decreased the viability of other human lung cancer cells (A549, CL1-0, H520, and H661) in a dose-dependent manner (Supplementary Fig. S2).

We further examined whether ACEE induces apoptosis in lung cancer cells by using flow cytometry-based detection of annexin V-positive cells. As shown in Fig. 1B, ACEE treatment for 24 h induced apoptosis in a dose-dependent manner in LLC cells (Fig. 1B). Apoptosis marker proteins such as cleaved caspase-3 (active form) and its downstream substrate, poly-(ADP-ribose)-polymerase (PARP), were also assessed using Western blot analysis. As shown in Figs 1C and S1C, ACEE induced cleavage of full-length PARP (116 kDa) into its active form (89 kDa) in LLC and CL1-5 cells. The level of cleaved caspase-3 fragments also increased following treatment of LLC and CL1-5 cells with ACEE (Fig. 1C and Supplementary Fig. S1C). These results indicate that ACEE reduces the viability of lung cancer cells by inducing apoptosis.

### Involvement of Bcl-2 proteins in ACEE-induced apoptosis

Members of the Bcl-2 family of proteins are involved in regulation of cell survival; these proteins include Bcl-2, which possesses anti-apoptotic properties, and Bax, which induces apoptosis^18^. We therefore examined the level of Bcl-2 family proteins in response to ACEE treatment. As shown in Fig. 1C, ACEE treatment decreased Bcl-2 protein level in LLC cells, while this treatment increased slightly Bax level. In CL1-5 cells, ACEE used at a concentration 0.1% also reduced Bcl-2 and increased Bax (Supplementary Fig. S1C). Quantitative analysis of Bcl-2 and Bax protein using densitometry showed that the Bax/Bcl-2 ratio increased in a dose-dependent manner following ACEE treatment in LLC cells (Fig. 1D). Treatment of CL1-5 cells with 0.1% ACEE significantly increased the Bax/Bcl-2 ratio compared with control ethanol (Supplementary Fig. S1D). We conclude that ACEE induces apoptosis of lung cancer cells by modulating Bcl-2 and Bax protein levels.

### ACEE inhibits JAK2/STAT3 signaling pathway activation in LLC cells

**Figure 2:**
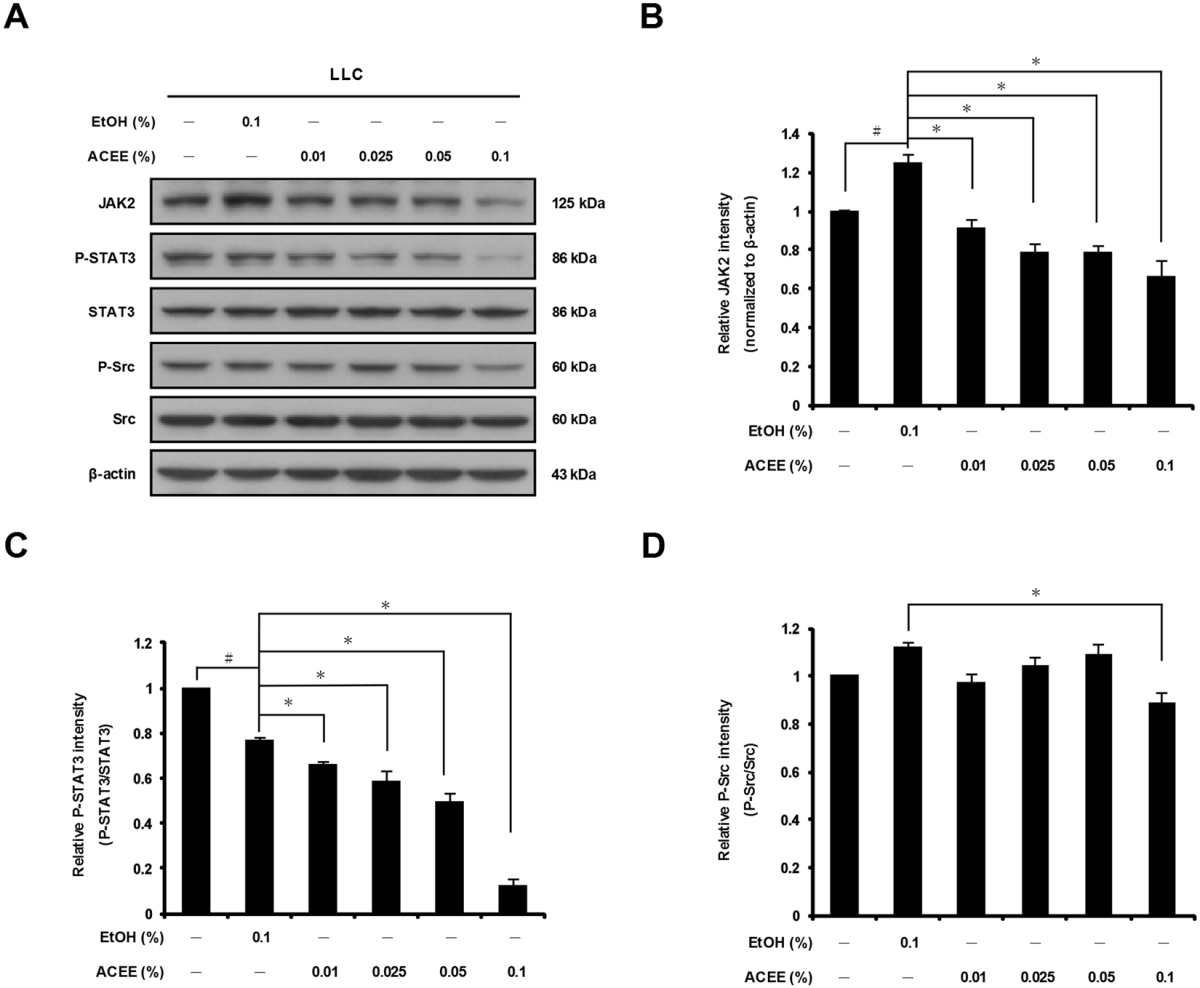
ACEE inhibits activation of the JAK2/STAT3 signaling pathway in LLC cells. (**A**) Cells were treated with ACEE for 6 h. Expression of JAK2, p-STAT3, STAT3, p-Src, Src, and β-actin was examined by Western blotting. (**B**) Relative band density of JAK2 was quantified by densitometry and normalized to β-actin. Ratios of P-STAT3 (**C**) and P-Src (**D**) normalized to the respective total protein level are shown. Data are presented as means ± SEM of three experiments preformed in duplicate. ^#^*P* < 0.05 versus untreated cells. *P < 0.05 versus control ethanol-treated cells.

Previous studies have shown that blockade of STAT3 activation in tumor cells induces apoptosis, inhibits cell proliferation and suppresses angiogenesis^3^. To investigate whether ACEE affects STAT3 activation in LLC cells, we measured the level of the STAT3 regulator JAK2 by using Western blotting. ACEE significantly reduced JAK2 protein level in LLC cells (Fig. 2A,B). ACEE treatment reduced the level of phosphorylated STAT3 (P-STAT3) in a dose-dependent manner, whereas total STAT3 protein level remained unchanged (Fig. 2A,C). ACEE treatment also reduced phosphorylated Src (P-Src) levels, especially at the concentration of 0.1%, while total Src level remained constant (Fig. 2A,D). These observations indicate that ACEE inhibits STAT3 signaling in LLC cells.

### ACEE affects survivin and p53 expression in LLC cells

**Figure 3:**
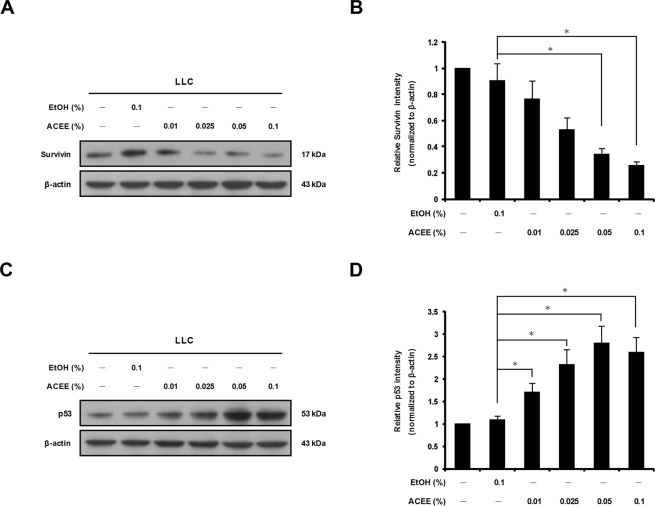
ACEE induces down-regulation of survivin and up-regulation of p53 in LLC cells. (**A**) Cells were treated with ACEE for 6 h, and protein expression of survivin was analyzed by Western blotting. (**B**) The relative band density of survivin was quantified by densitometry and normalized to β-actin. (**C**) Cells were treated with ACEE for 24 h, and Western blotting was performed to assess p53 protein expression. (**D**) Relative band density of p53 was quantified by densitometry and normalized to β-actin. Data are presented as means ± SEM of three experiments preformed in duplicate. *P < 0.05 versus control ethanol-treated cells.

The protein survivin, a member of the inhibitor of apoptosis (IAP) family, is a target gene of the transcription factor STAT3 and is known to be crucial for the proliferation and survival of cancer cells. A previous study showed that inhibition of STAT3, which is constitutively activated in gastric cancer cells, represses survivin expression^19^. We therefore measured survivin expression in LLC cells after ACEE treatment. As shown in Fig. 3A,B, survivin protein level decreased in a dose-dependent manner in LLC cells treated with ACEE for 6 h. Another study showed that inhibition of STAT3 activation up-regulates p53 expression in cancer cells, leading to p53-induced apoptosis^20^. As shown in Fig. 3C,D, we observed that ACEE treatment for 24 h increased p53 protein level compared with control ethanol. These results suggest that ACEE may produce anti-cancer effects by affecting expression of survivin and p53.

### ACEE inhibits tumor growth and lung metastasis *in vivo*

**Figure 4:**
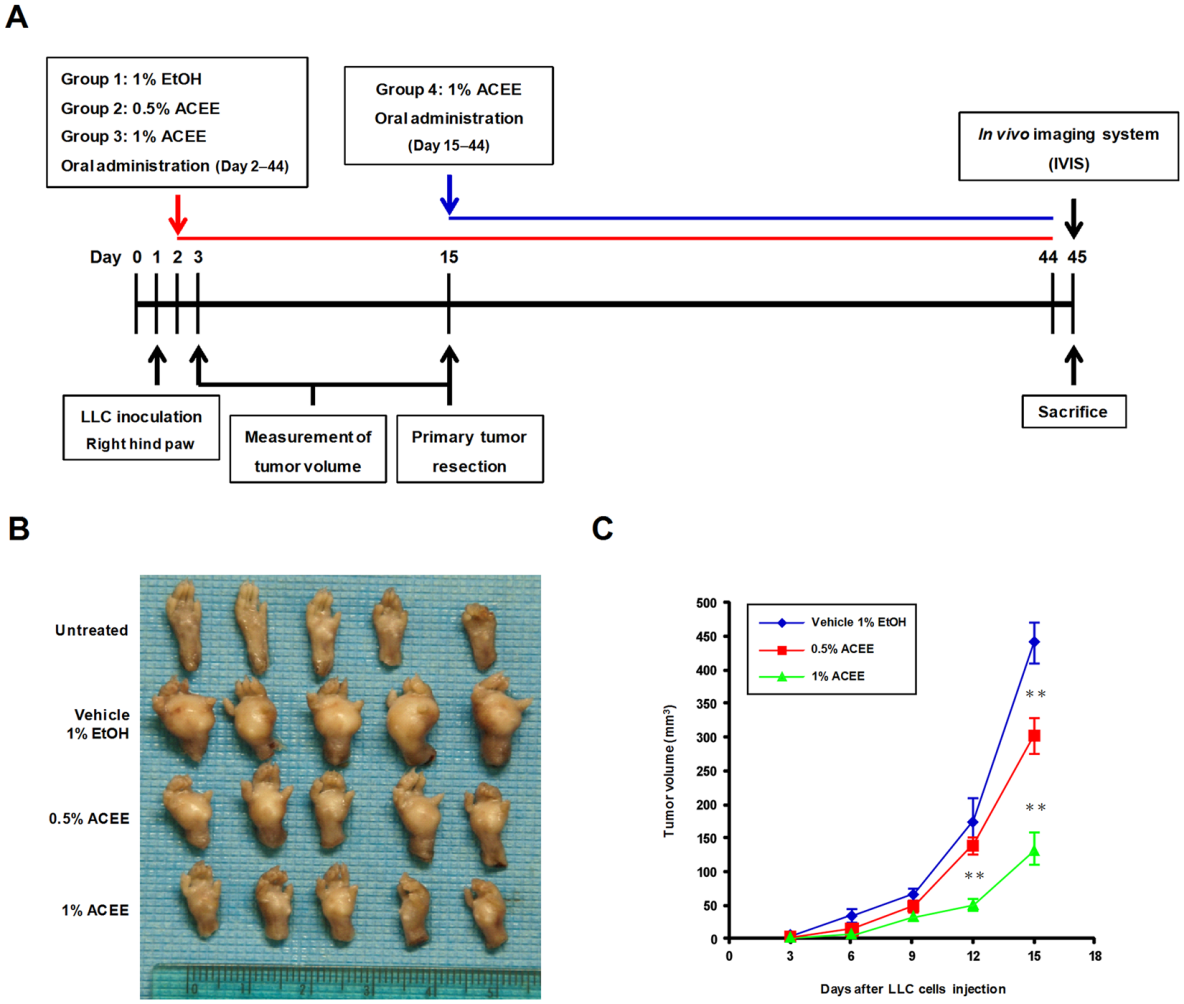
ACEE inhibits tumor growth in LLC tumor-bearing mice. (**A**) Schedule of *in vivo* experiments. LLC-LT cells were inoculated into the right hind paw of C57BL/6 mice. ACEE (0.5 and 1%) was orally administered five times per week. Primary tumors were resected on day 15, and mice were sacrificed on day 45. (**B**) Representative images of primary tumors for the vehicle control and ACEE-treated groups. (**C**) Volume (mm^3^) of developing LLC paw tumors in vehicle and ACEE-treated mice was assessed by using a digital caliper on day 3, 6, 9, 12 and 15. Data are presented as means ± SEM (n = 5 in each group). **P < 0.01 versus the vehicle group.

We used an animal model of LLC-induced allograft tumors to evaluate the anticancer effects of ACEE *in vivo* (Fig. 4A). In this animal model, the use of LLC-LT cells expressing luciferase allowed bioluminescence-based detection of tumor cells *in vivo*. LLC-LT cells were transplanted into the right hind paw of C57BL/6 mice and tumors formed locally before producing metastases in the lungs. Notably, ACEE treatment at 0.5 and 1% considerably reduced primary tumor growth at the site of injection (Fig. 4B). ACEE treatment reduced tumor volume in a dose-dependent manner compared with control ethanol (Fig. 4C). ACEE produced no apparent side effects on blood biochemical parameters (Table 1) or body weight (data not shown). We also examined the effects of ACEE on the growth of xenograft tumors produced by subcutaneous inoculation of human A549 lung cancer cells into nude mice. As shown in Supplementary Fig. S3A,B, ACEE treatment reduced tumor size and volume in a dose-dependent manner compared with the control group treated with vehicle 1% ethanol. Haematoxylin and eosin (H&E) staining of tumor tissues revealed that, while no sign of tissue damage was detected for the mice treated with vehicle ethanol, ACEE treatment increased tissue damage and necrosis in a dose-dependent manner in tumor tissues compared with the control group (Supplementary Fig. S3C).

**Table 1:** Absence of effects of ACEE on biochemical parameters in mouse serum.

Parameters	Vehicle control	0.5% ACEE	*P-*values	1% ACEE	*P-*values
AST (U/l)	167.0 ± 16.36	158.0 ± 13.86	0.7873	141.1 ± 28.72	0.5276
ALT (U/l)	35.7 ± 3.94	38.0 ± 4.09	0.3785	36.0 ± 1.55	0.9857
TP (g/dl)	5.1 ± 0.04	5.0 ± 0.12	0.2943	5.0 ± 0.03	0.0705
ALB (g/dl)	3.1 ± 0.04	3.1 ± 0.07	0.6072	3.0 ± 0.05	0.0993
BUN (mg/dl)	28.6 ± 1.78	28.3 ± 2.95	0.9049	30.4 ± 3.35	0.7141
CREA (mg/dl)	0.2 ± 0.01	0.2 ± 0.01	0.9156	0.2 ± 0.01	0.5415
T-CHO (mg/dl)	80.3 ± 2.09	77.8 ± 0.31	0.3703	78.1 ± 2.90	0.6179
TG (mg/dl)	19.9 ± 0.17	23.2 ± 7.35	0.6946	18.8 ± 2.70	0.7178
Ca (mg/dl)	7.6 ± 0.25	8.3 ± 0.32	0.2778	8.2 ± 0.19	0.0519
P (mg/dl)	6.5 ± 0.15	7.0 ± 0.29	0.3076	6.8 ± 0.03	0.1732
Mg (mg/dl)	2.2 ± 0.07	2.1 ± 0.06	0.2697	2.1 ± 0.03	0.2254

Data are expressed as means ± SEM (n = 5). No significant statistical difference was noted between the biochemical parameters of control and ACEE-treated groups (*P* > 0.05). AST: aspartate transaminase; ALT: alanine transaminase; TP: total proteins; ALB: albumin; BUN: blood urea nitrogen; CREA: creatinine; T-CHO: total cholesterol; TG: triglycerides; Ca: calcium; P: phosphorus; Mg: magnesium.

**Figure 5:**
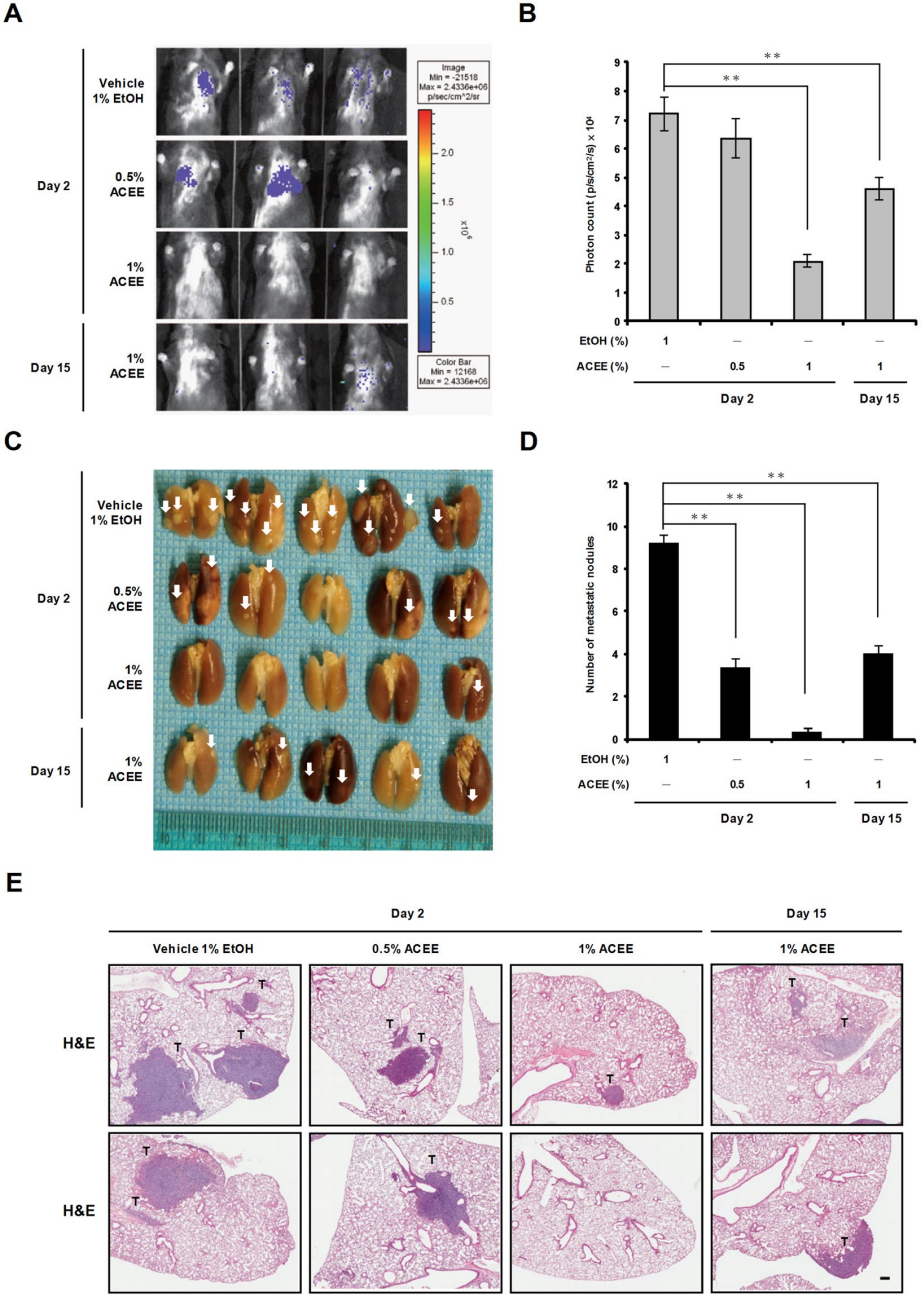
ACEE inhibits lung metastasis of LLC cells *in vivo*. (**A**) Bioluminescence images of control and ACEE-treated mice were obtained using the Bioluminescent IVIS Imaging System. The color scale depicts photon flux (photons/sec/cm^2^/steradian). (**B**) Magnitude of bioluminescent signal (p/s/cm^2^/s) representing tumor growth *in vivo* on day 45. (**C**) Lung metastatic nodules were visualized to show the inhibitory effects of ACEE on LLC tumor. White arrowheads indicate metastatic nodules. (**D**) Number of lung metastatic nodules formed by LLC cells in each group. (**E**) Representative lung tissue sections were stained with H&E. Tumor tissues are marked with “T”. Scale bar = 200 μm. Data are presented as means ± SEM (n = 5). **P < 0.01 versus the vehicle group.

We further examined whether ACEE treatment reduces the occurrence of lung metastases in this animal model. Bioluminescence imaging *in vivo* showed that ACEE treatment significantly reduced photon counts from the body surface of mice (Fig. 5A,B). Moreover, ACEE administered at 0.5 and 1% significantly reduced the number of lung metastatic nodules compared with the control group (Fig. 5C,D). As expected, ACEE treatment (1%) starting on day 2 produced higher anti-metastatic activity than treatment starting on day 15 (Fig. 5A–D). The number and size of micrometastatic nodules per field was also significantly lower in ACEE-treated groups compared with the control group, as assessed in H&E-stained lung tissues (Fig. 5E). These results reveal that ACEE produces antitumor and anti-metastatic effects in animals.

### ACEE induces cancer cell apoptosis *in vivo*

**Figure 6:**
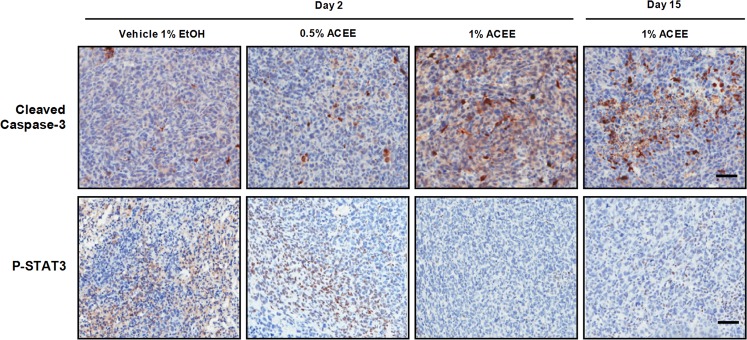
ACEE suppresses lung tumor growth *in vivo* by inducing cleavage of caspase-3 and by reducing P-STAT3 level. Immunohistochemistry staining was used to examine cleaved caspase-3 and P-STAT3 levels in mouse tumor tissues. Representative images of LLC cells that stained positive for cleaved caspase-3 or P-STAT3 in tumor sections obtained from control vehicle and ACEE-treated mice on day 45. Scale bar = 100 μm.

To determine whether ACEE induces tumor cell apoptosis *in vivo*, we examined the effect of ACEE on the level of cleaved caspase-3 in LLC tumor allografts. Based on immunohistochemistry analysis, cleaved caspase-3 level was significantly increased in ACEE-treated groups compared with the control group (Fig. 6). In addition, immunohistochemistry analysis of ACEE-treated LLC tumor allografts showed that the expression level of P-STAT3 was significantly reduced in ACEE-treated tumor sections (Fig. 6), indicating that ACEE induces apoptosis and reduces P-STAT3 level in cancer cells *in vivo*.

## Discussion

Numerous studies have shown that the JAK2/STAT3 signaling pathway, which regulates many cellular processes including proliferation, survival, metastasis and angiogenesis, is constitutively activated in various tumor cell lines and primary tumors^3,5^. The JAK2/STAT3 signaling pathway therefore represents a potential target for cancer therapy^21^. In the present study, we observed that ACEE induces apoptosis in lung cancer cells and reduces tumor growth and metastasis in an animal model of allograft tumor in mice. Notably, ACEE significantly reduces the expression of JAK2 and P-STAT3 in LLC cells, in addition to reducing P-STAT3 level in murine allograft tumors. These results suggest that ACEE may suppress tumor growth by inhibiting the JAK2/STAT3 signaling pathway.

Several anti-apoptosis proteins such as survivin and Bcl-2, which are known to be crucial for tumor survival, represent targets of the transcription factor STAT3 and are down-regulated as a consequence of STAT3 inhibition^22^. In cancer cells, constitutively activated STAT3 may inhibit p53 expression by binding to the p53 promoter^20^, thereby preventing p53-mediated apoptosis and contributing to cell survival. As a pro-apoptotic transcription factor, the p53 protein also down-regulates Bcl-2 and up-regulates Bax, thereby affecting the Bcl-2/Bax ratio and favoring apoptosis^23^. In the present study, we observed that ACEE treatment reduces expression of the STAT3-modulated anti-apoptotic proteins Bcl-2 and survivin in LLC cells, in addition to increasing expression of the pro-apoptotic proteins Bax and p53. ACEE also induced cleavage of apoptosis markers such as caspase-3 and PARP in LLC cells. A previous study reported that antrocin, a sesquiterpene lactone isolated from *A. cinnamomea*, induces apoptosis in human bladder cancer cells via a Bcl-2-dependent pathway and caspase-3 activation^24^. These observations indicate that ACEE induces apoptosis in lung cancer cells by inhibiting the JAK2/STAT3 signaling pathway and by inducing apoptosis via modulation of Bax/Bcl-2, caspase-3 and PARP.

We demonstrated that ACEE produces anti-tumor activity on lung cancer *in vivo*. Oral administration of ACEE in a mouse allograft tumor model significantly reduces tumor volume and increases cleavage of caspase-3 in tumor tissues. Our results indicate that ACEE significantly reduces the number of lung tumor nodules in LLC-bearing mice, indicating that ACEE may reduce tumor metastases to the lungs. The anti-cancer and anti-metastasis activities of ACEE may be produced by triterpenoids and the lanostanoid compound pinicolol B which have been identified in the extract studied here (triterpenoids, 34.3%, w/w; pinicolol B, 91 ppm; see Methods; more information about the chemical analysis of ACEE is also found in our previous study)^25^. These triterpenoid and lanostenoid compounds have been shown to produce anticancer effects in previous studies^26–28^.

In conclusion, the present study reveals that an ethanol extract of *A. cinnamomea* mycelium effectively inhibits tumor growth and metastasis by inducing apoptosis in lung cancer cells and LLC tumor allografts in mice. The anti-cancer effects of ACEE in lung cancer cells are mediated at least in part by down-regulation of the JAK2/STAT3 signaling pathway. These results suggest that ACEE represents a potential candidate for lung cancer treatment and the isolation of anticancer compounds.

## Methods

### Chemical reagents

Cell culture media and chemical reagents including Dulbecco’s modified Eagle’s medium (DMEM), minimum essential medium (MEM), Opti-MEM, Roswell Park Memorial Institute (RPMI) 1640, sodium pyruvate, and antibiotics were obtained commercially (Life Technologies, Grand Island, NY, USA). Fetal bovine serum (FBS) was used according to the manufacturer’s instructions (HyClone, Logan, UT, USA). Commercial antibodies were used to detect Src, phosphorylated-Src (Tyr416), STAT3, phosphorylated-STAT3 (Tyr705), JAK2, cleaved caspase-3, cleaved PARP, full-length PARP (Cell Signaling Technology, Beverly, MA, USA), Bax, Bcl-2, p53, survivin, and β-actin (Santa Cruz Biotechnology, Dallas, TX, USA). Antibody detection was done using goat anti-rabbit and anti-mouse antibodies coupled to horseradish peroxidase (HRP; Santa Cruz Biotechnology).

### Cell culture

Cell lines including mouse Lewis lung carcinoma cells (LLC; BCRC-60050), human non-cancerous fetal lung fibroblasts (MRC-5; BCRC-60023), human lung adenocarcinoma cells (A549; BCRC-60074), human lung squamous cell carcinoma cells (NCI-H520; BCRC-60124), and human large cell lung cancer cells (NCI-H661; BCRC-60125) were purchased locally (Bioresource Collection and Research Center, BCRC, Food Industry Research and Development Institute, Hsinchu, Taiwan). Human lung adenocarcinoma cell lines (CL1 sublines, the less invasive subline CL1-0 and the more invasive subline CL1-5) were kindly provided by Dr. C. M. Chen (National Chung Hsing University, Taichung, Taiwan). Human embryonic kidney cells (HEK-293T; CRL-3216) were obtained from the American Type Culture Collection (Manassas, VA, USA). A549, LLC, CL1-0, CL1-5 and HEK-293 cell lines were grown in DMEM, while H520 and H661 cells were cultured in RPMI 1640 medium. Both cell culture media were supplemented with FBS (10%, v/v), sodium pyruvate (1 mM), penicillin (100 units/ml) and streptomycin (100 μg/ml). MRC-5 cells, which were used as control non-cancerous lung cells as before^29–31^, were cultured in MEM containing FBS, sodium pyruvate, penicillin and streptomycin as above. All cells were incubated at 37 °C in standard cell culture conditions.

### Mycelium extract

*A. cinnamomea* mycelium was isolated by Chang Gung Biotechnology (Taipei, Taiwan) and identified by DNA analysis of 5.8 S rDNA and internal transcribed spacer-1 and 2 (ITS-1 and ITS-2). DNA sequences were compared with a type strain of the BCRC database (AJ496398). ACEE was prepared as before^8^, with minor modifications as described below to increase the concentration of the extract. *A. cinnamomea* mycelium (~400 g) was mixed with 10 liters of ethanol (95%, v/v). Following agitation for 1 h at 80 °C, the solution was centrifugated (5,900 *g*) 30 min at room temperature. A vacuum concentrator was used to concentrate the supernatant to obtain a sample of approximately 80 g. After centrifugation (5,900 *g*, 30 min), the supernatant corresponding to ACEE was collected and stored at 4 °C.

### Triterpenoid and pinicolol B quantification

Triterpenoids were quantified as before^32^. Pinicolol B was quantified using high-performance liquid chromatography (HPLC) with a Cosmosil 5C18-MS-II column (4.6 × 250 mm, 5 μm). The mobile phase consisted of 0.009% phosphoric acid (solution A) and acetonitrile (solution B). Elution was started with a mobile phase containing 70% solution A and 30% solution B. The gradient of solutions A and B was changed to 53/47 from 0–110 min, and to 0/100 from 110–170 min. A flow rate of 1 ml/min and a photodiode detector was used at 243 nm.

### Cell viability assay

A commercial MTT kit was used to monitor cell viability (Sigma-Aldrich, St. Louis, MO, USA). Briefly, LLC (1.5 × 10^4^ cells/well), A549, CL1-0, CL1-5 (1 × 10^4^ cells/well), H520 (4 × 10^4^ cells/well), H661 (6 × 10^3^ cells/well), and MRC-5 (2 × 10^4^ cells/well) cells were cultured in 96-well plates. After 24 h, the cells were treated with ACEE for 24 h. The cell culture medium was removed and replaced with 5 mg/ml of MTT for 4 h. One hundred μl of MTT solubilization solution was added to each well, followed by mixing in a shaker for 10 min. Absorbance was measured at 570 nm using the VersaMax microplate reader (Molecular Devices, Sunnyvale, CA, USA). Cell viability percentage was calculated as the ratio of surviving cells in the ACEE-treated group divided by that of the control group.

### Apoptosis assay

Apoptosis was monitored using the Annexin V-FITC Apoptosis Detection Kit (BioVision, Mountain View, CA, USA) as previously described^33^. Briefly, LLC (5 × 10^5^ cells/well) cells were cultured in 6-well plates. After 24 h, the cells were treated with ACEE for 24 h. Both floating and adherent cells were collected and washed with PBS. Apoptosis was examined by flow cytometry (FACSCalibur, BD Biosciences, San Jose, CA, USA) based on the manufacturer’s instructions.

### Establishment of LLC-LT cells

Virus stocks were prepared by co-transfecting the pLenti-LucDsRed (LT) plasmid with three packaging plasmids, pMDLg/pRRE, CMV-VSVG and RSV-Rev (Addgene, Cambridge, MA, USA), into 293 T cells. Supernatants containing viral particles were harvested 36–48 h later, filtered and centrifuged at 20,000 *g* for 90 min. Viral titer was determined by the end-point dilution method by counting the number of infected 293 T red cells at 100× magnification under a fluorescence microscope (Nikon, Tokyo, Japan) 96 h after infection. The titer of transducing units (TU) was computed as follows: TU/ml = (the numbers of red fluorescent cells) × (dilution factor)/(volume of virus solution). LLC cells were seeded in 12-well plates and the cells were transduced with an equal amount of LT virus particles. Stably transducted cells were designated as LLC-LT.

### Lung cancer animal model

Animal experiments were approved by the Institutional Animal Care and Use Committee (Chang Gung University). Experiments were done according to the guidelines. Commercial C57BL/6 mice (eight-week old, male, ~23.5 g; National Laboratory Animal Center, Taipei, Taiwan) were maintained in a 12 h light/12 h dark cycle with access to food and water at all time. After seven days, the animals were divided into four groups (n = 5/group). Treatments were as follows: group 1: 1% ethanol starting on day 2; group 2: 0.5% ACEE starting on day 2; group 3: 1% ACEE starting on day 2; group 4: 1% ACEE starting on day 15. On day 1, Opti-MEM medium (50 μl) containing 2.5 × 10^5^ LLC-LT cells was inoculated into right hind paws. One day after cell injection, ACEE at concentrations of 0.5% or 1% (dissolved in 0.1 ml PBS) and the 1% ethanol vehicle (dissolved in 0.1 ml PBS) were given by oral gavage (5 times/week). Tumor size was determined with calipers every three days starting on day 3. Tumor volume was calculated using the following formula: tumor volume (mm^3^) = larger diameter (mm) × small diameter (mm^2^)/2. On day 15, primary solid tumors were resected. On day 45, all mice were sacrificed; blood and lung tissues were harvested for further analysis. Metastasized pulmonary nodules were counted using a stereomicroscope.

### *In vivo* tumor xenograft experiments

Eight-week-old male BALB/c nude mice (18 to 22 g) were purchased from the National Laboratory Animal Center. Exponentially growing A549 cells were mixed at a 1:1 ratio with Matrigel (BD Biosciences), and a 100 μl suspension containing 2 × 10^6^ cells was injected subcutaneously in the right flank of each mouse. After 12 days, mice were divided to form four groups (n = 8 mice per group). The animals were treated by oral gavage with 0.5, 1 or 2% ACEE in 0.1 ml of PBS five days per week. Mice in the control group were treated as described above. Tumor volume was measured using calipers on day 12, 22, 29, 36, 43, and 52. The experiment was terminated 52 days after tumor cell inoculation. At the time of sacrifice, tumors were excised and photographed. Tumor samples were also fixed in 4% paraformaldehyde and embedded in paraffin for histological analysis.

### Bioluminescence imaging *in vivo*

Bioluminescence imaging was performed using the IVIS Imaging System (Xenogen, Alameda, CA, USA). D-luciferin (Promega, Madison, WI, USA) was dissolved in PBS (15 g/l) and injected intraperitoneally at a dose of 10 μl/g of body weight, 40 min before observation. Mice were anesthetized using a 2% isoflurane/oxygen mixture and placed in the imaging chamber. A region of interest was drawn for each tumor, and the signal was calculated based on the number of photons emitted from the body surface (photons/sec/cm^2^/steradian). Photons emitted from specific regions were quantified using the Living Image Software (Xenogen).

### Histopathology and immunohistochemistry

Lung tissues were collected from sacrificed mice. Tissues were fixed with 4% paraformaldehyde and embedded using paraffin. Paraffin-embedded sections (4 µm) from successive cuts were obtained using a microtome. Deparaffinized tissue slices were treated with hematoxylin and eosin (H&E) stains using a standard protocol. Paraffin-embedded tissues were submitted to immunohistochemistry analysis using a commercial detection system (EnVision Detection Systems, Dako, Glostrup, Denmark) based on the manufacturer’s guidelines. Sections were boiled 10 min in a solution of sodium citrate (10 mM, pH 6.0) to expose antigens, incubated with antibodies against cleaved caspase-3 or p-STAT3 overnight at 4 °C, followed by incubation with a secondary antibody for 1 h at 37 °C. Sections were stained with freshly prepared DAB substrate (Dako). Counterstaining was done with hematoxylin. Sections were dehydrated and placed on glass slides. Images were obtained with a commercial system (HistoFAXS, Tissue Gnostics, Vienna, Austria). Immunostaining was observed in a blind manner by two experienced pathologists.

### Western blotting

Protocols used for protein extraction and Western blotting were described before^25^. Briefly, equal amounts of proteins were loaded and separated onto a 12% SDS-PAGE. Transfer was done to PVDF membranes (Millipore, Billerica, MA, USA) that were blocked with non-fat milk (5%). Blocking was done at room temperature for 1 h with agitation. Primary antibody incubation was done overnight at 4 °C. Membranes were washed and treated with secondary antibody at room temperature for 1 h. Protein signal was detected using chemiluminescence (Millipore). Band intensity was normalized to β-actin used as a loading control.

### Statistics

The experimental results shown represent means ± standard error of the mean (SEM). Experiments were repeated at least three times using duplicate specimens for each treatment. The results of multiple groups were compared using one-way analysis of variance (ANOVA) and Dunnett’s post hoc test. The means of two groups were compared with a two-tailed Student’s *t*-test. *P* values of less than 0.05 were considered statistically significant.

## Supplementary information

Supplementary Information
