# Supplementary material for: Antrodia cinnamomea induces anti-tumor activity by inhibiting the STAT3 signaling pathway in lung cancer cells
Source: Sci Rep. 2019 Mar 26;9:5145. doi: 10.1038/s41598-019-41653-9 (PMC6435735; doi:10.1038/s41598-019-41653-9)
Supplement: Supplementary file 1 — Supplementary Information [file 41598_2019_41653_MOESM1_ESM.pdf]

Supplementary Information

Antrodia cinnamomea induces anti-tumor activity by inhibiting the STAT3 signaling pathway in lung cancer cells

Tsung-Teng Huang[1,2,3], Ying-Wei Lan[1,4], Chuan-Mu Chen[5,6], Yun-Fei Ko[3,7,8], David M. Ojcius[2,3,9], Jan Martel[2,3], John D. Young[2,3,7,8,10] and Kowit-Yu Chong[1,4,11,12]

[1]Department of Medical Biotechnology and Laboratory Sciences, College of Medicine, Chang Gung University, Taoyuan 33302, Taiwan.

[2]Center for Molecular and Clinical Immunology, College of Medicine, Chang Gung University, Taoyuan 33302, Taiwan.

[3]Chang Gung Immunology Consortium, Chang Gung Memorial Hospital, Linkou, Taoyuan 33305, Taiwan.

[4]Graduate Institute of Biomedical Sciences, Division of Biotechnology, College of Medicine, Chang Gung University, Taoyuan 33302, Taiwan.

[5]Department of Life Sciences, and Ph.D. Program in Translational Medicine, National Chung Hsing University, Taichung 402, Taiwan.

[6]The iEGG and Animal Biotechnology Center, National Chung Hsing University, Taichung 402, Taiwan.

[7]Chang Gung Biotechnology Corporation, Taipei 10508, Taiwan.

[8]Biochemical Engineering Research Center, Ming Chi University of Technology, New Taipei City 24301, Taiwan.

[9]Department of Biomedical Sciences, University of the Pacific, Arthur Dugoni School of Dentistry, San Francisco, CA 94103, USA.

[10]Laboratory of Cellular Physiology and Immunology, Rockefeller University, New York, NY 10021, USA.

[11]Department of Family Medicine, Chang Gung Memorial Hospital, Linkou, Taoyuan 33305, Taiwan.

[12]Department of Preclinical Sciences, Faculty of Medicine and Health Sciences and Centre for Stem Cell Research, Universiti Tunku Abdul Rahman, Kajang 43000, Selangor, Malaysia.

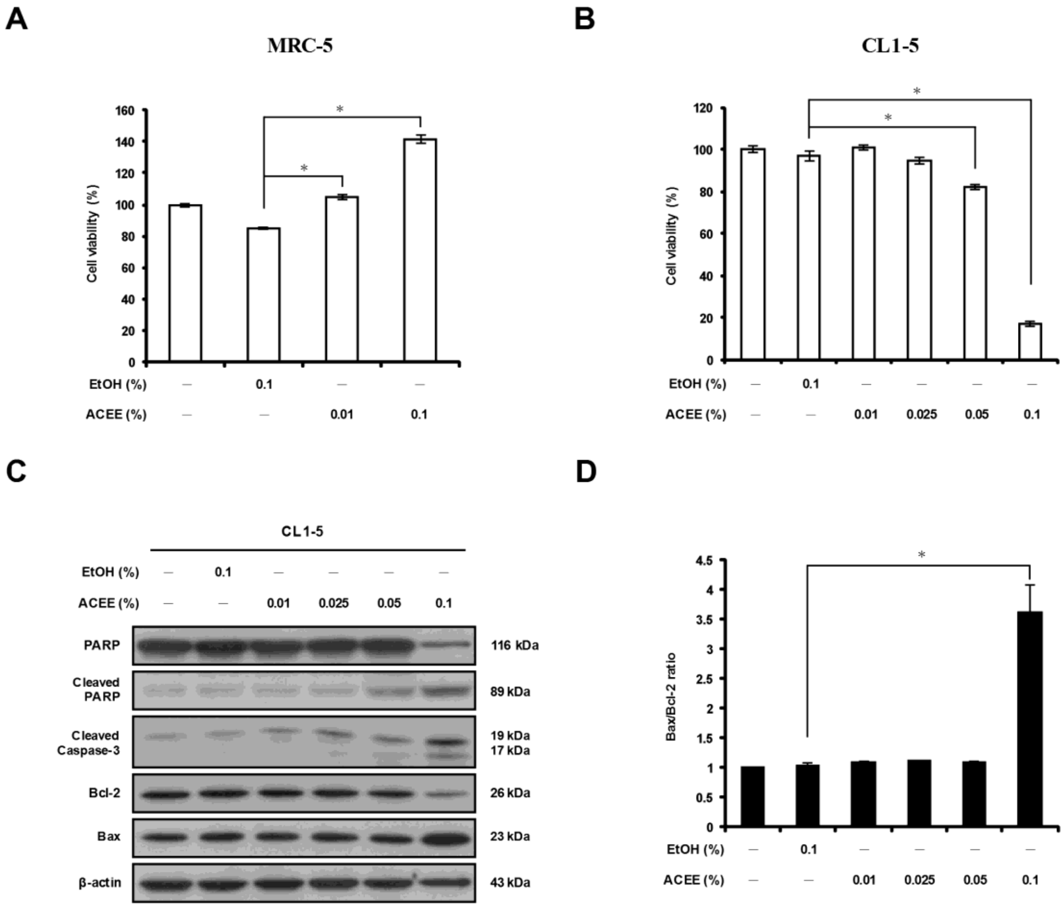

Figure S1. Effects of ACEE on cell viability and apoptosis markers in human fibroblasts and lung cancer cells. (**A**) Human MRC-5 fetal lung fibroblasts and (**B**) CL1-5 human lung cancer cells were treated with ACEE for 24 h, and cell viability was monitored using the MTT assay. (**C**) Western blotting of PARP, cleaved PARP, cleaved caspase-3, Bcl-2 and Bax in ACEE-treated CL1-5cells. β-actin was used as an internal control. (**D**) Relative Bax/Bcl-2 protein ratio in ACEE-treated CL1-5 cells determined using densitometry. Data are presented as means ± SEM of three experiments performed in duplicate. *$P < 0.05$ versus control ethanol-treated cells.

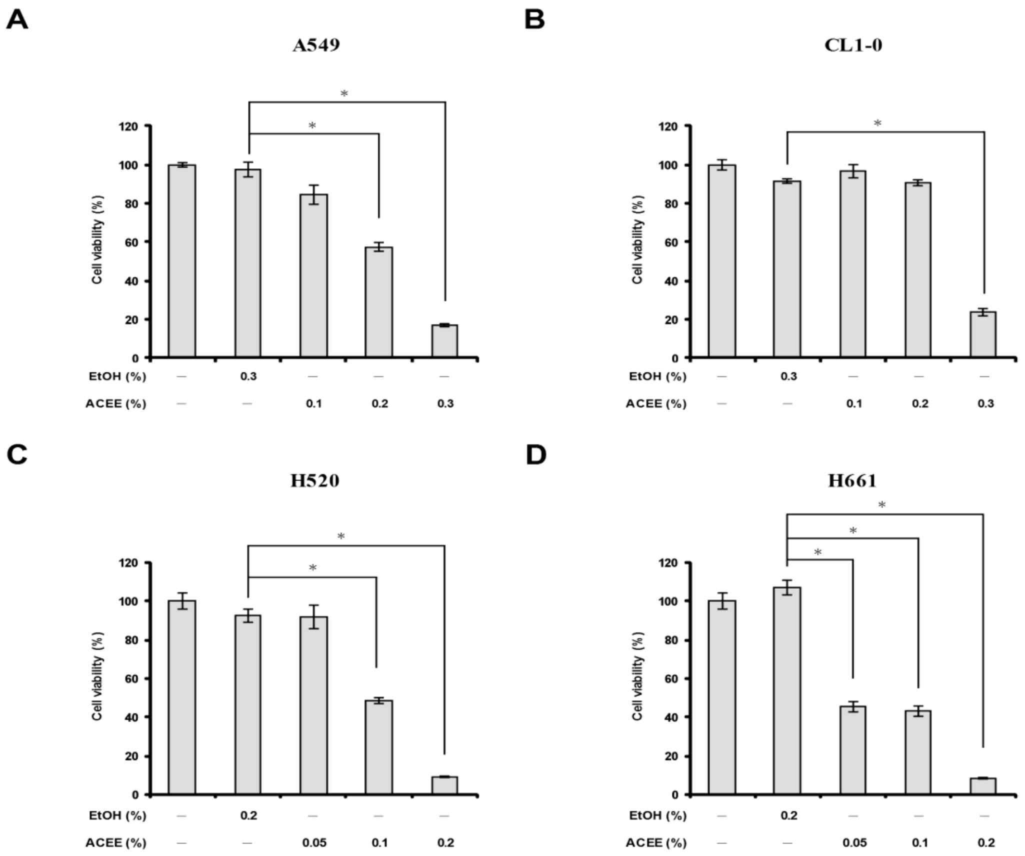

Figure S2. ACEE inhibits viability of human lung cancer cells. (**A**) A549, (**B**) CL1-0, (**C**) H520, and (**D**) H661 cells were treated with ACEE at various concentrations as indicated for 24 h. Cell viability was examined using the MTT assay. Data are presented as means ± SEM of three experiments performed in duplicate. *$P < 0.05$ versus control ethanol-treated cells.

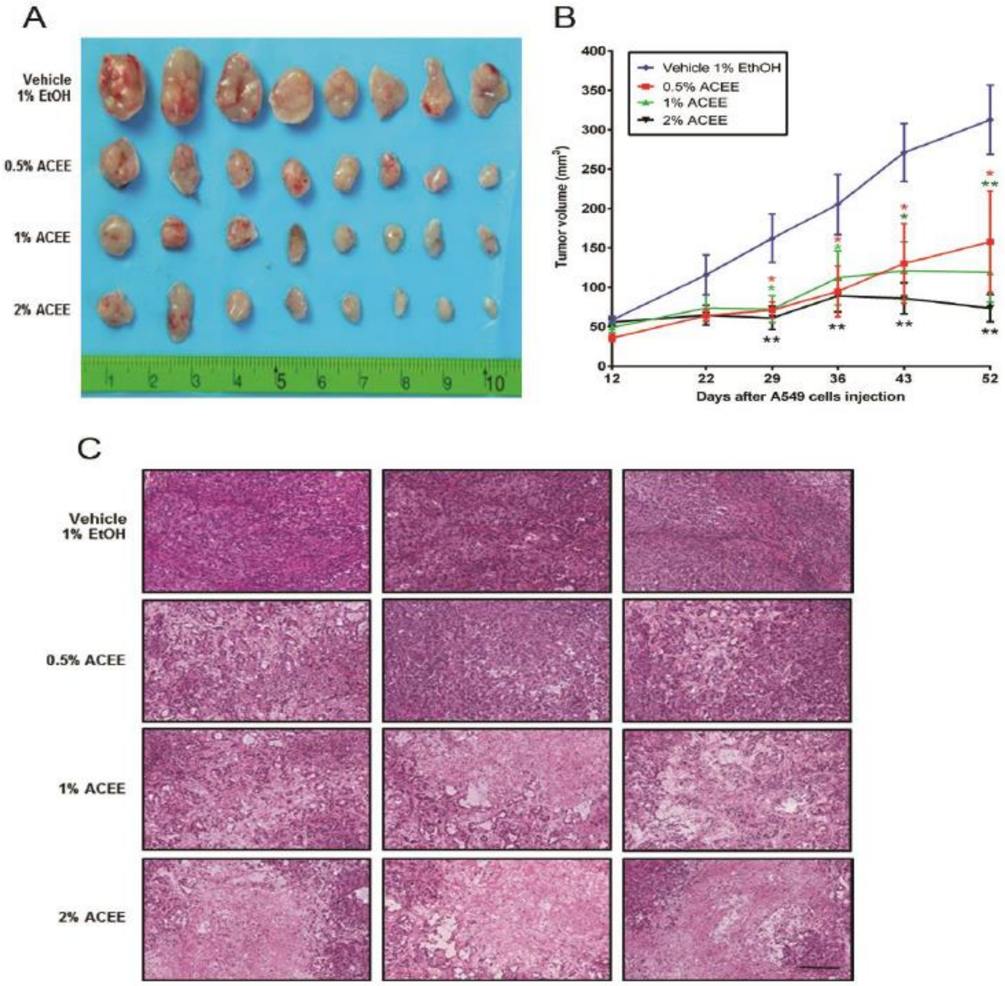

Figure S3. ACEE inhibits the growth of tumor xenografts in nude mice. A549 cells (2×10^6 cells/mice) were injected subcutaneously into the right flank of BALB/c nude mice. After 12 days, the animals were randomized into four groups: group I was treated with 1% ethanol as a vehicle control; group II was treated with 0.5% ACEE; group III was treated with 1% ACEE; and group IV was treated with 2% ACEE. Treatment with the vehicle (1% ethanol dissolved in 0.1 ml of PBS) or ACEE (0.5–2% ACEE dissolved in 0.1 ml of PBS) was administered by oral gavage five days per week. (**A**) Images of tumors excised from nude mice on day 52 (n = 8 mice/group). (**B**) Tumor volume measurements during treatment with ACEE or control vehicle. (**C**) Histological analysis of tumor tissues stained with H&E. Scale bar = 200 µm. Data are presented as means ± SEM (n = 8). $^{**}P < 0.01$ versus the vehicle group.

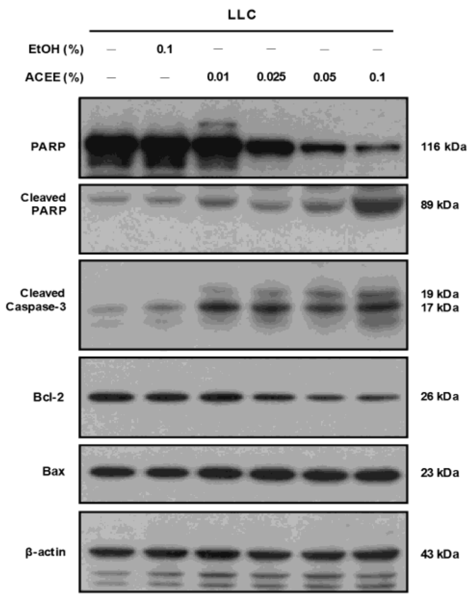

Figure S4. Full-width Western blot membranes of Fig. 1C.

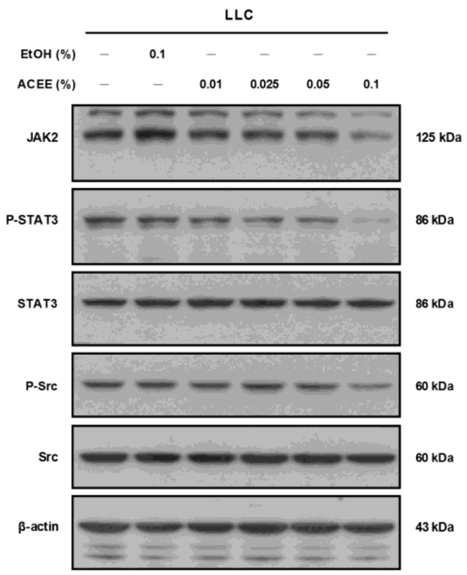

Figure S5. Full-width Western blot membranes of Fig. 2A.

Figure 3A

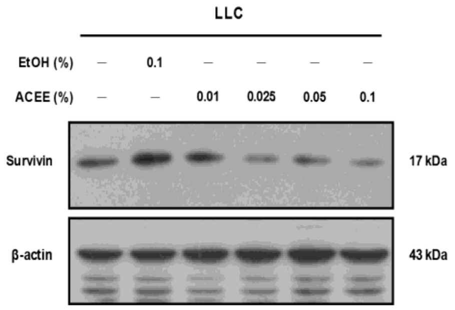

Figure 3C

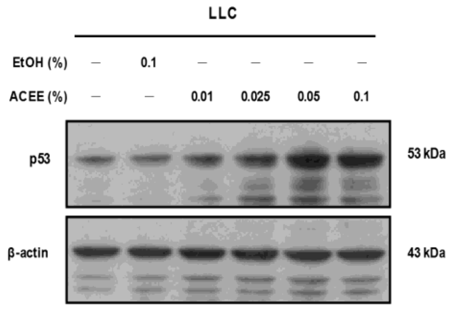

Figure S6. Full-width membranes of the original blots used in Fig. 3A and C.

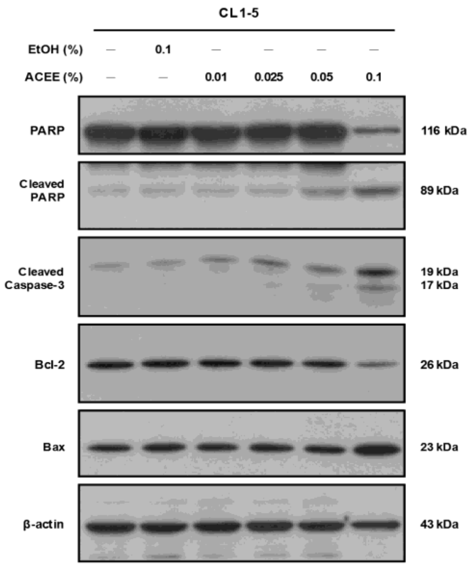

Figure S7. Full-width Western blot membranes used in Fig. S1C.
